# Simultaneous Prostate and Bladder Cancer with Collision Lymph Node Metastasis: A Case Report and Literature Review

**DOI:** 10.3390/medicina60091482

**Published:** 2024-09-11

**Authors:** Maximilian Buzoianu, Iulia Andras, Lorin Giurgiu, Claudia Florentina Militaru, Andrei Popa, Emanuel Darius Căta, Paul Alexandru Medan, Marius Cosmin Apetrei, Catalina Bungărdean, Maria Bungărdean, Nicolae Crișan

**Affiliations:** 1 Urology Department, Iuliu Hatieganu University of Medicine and Pharmacy, 400487 Cluj-Napoca, Romania; lorin.giurgiu@gmail.com (L.G.); popaandrei.dr@gmail.com (A.P.); emanuelcata@yahoo.com (E.D.C.); medan.paul@gmail.com (P.A.M.); apetrei.marius_cosmin@yahoo.com (M.C.A.); drnicolaecrisan@gmail.com (N.C.); 2 Pharmacology Department, Iuliu Hatieganu University of Medicine and Pharmacy, 400487 Cluj-Napoca, Romania; claudiamilitaru@yahoo.com; 3 Pathology Department, Clinical Municipal Hospital, 400487 Cluj-Napoca, Romania; catalina.bungardean@yahoo.com; 4 Pathology Department, Iuliu Hatieganu University of Medicine and Pharmacy, 400487 Cluj-Napoca, Romania; maria.bungardean@yahoo.com

**Keywords:** collision metastasis, bladder cancer, urothelial carcinoma, prostatic adenocarcinoma

## Abstract

Synchronous prostatic adenocarcinoma found in patients with muscle-invasive bladder cancer (MIBC) that undergo radical cistoprostatectomy is not uncommon. Nonetheless, the occurrence of collision metastasis, where both prostate cancer and bladder cancer involve the same lymph node, is exceptionally uncommon, with few cases being reported in the literature. We present a case of a 65-year-old patient diagnosed with MIBC who underwent laparoscopic radical cistoprostatectomy with extended lymph node dissection and intracorporeal ileal conduit. The final pathology revealed urothelial carcinoma pT3bN1 as well as prostatic adenocarcinoma pT3bN1. One lymph node presented metastasis from both bladder cancer and prostate cancer.

## 1. Introduction

Bladder cancer (BC) is the tenth most common malignancy, with a higher incidence in men, where it ranks as the sixth most common tumor [1]. In 2020, there were 573,000 new cases of BC and 213,000 deaths reported globally [2]. The majority of cases are urothelial tumors, but other histological types, such as adenocarcinoma, squamous cell carcinoma, neuroendocrine carcinoma, epidermoid carcinoma, and lymphoma, are also seen [3]. Approximately 75% of bladder cancers are non-muscle-invasive tumors, which can be managed conservatively (through transurethral resection of the tumor and intravesical chemotherapy/BCG) with a good prognosis. However, 25% of cases are muscle-invasive bladder cancers (MIBC), which have a poorer prognosis and require systemic chemotherapy and/or immunotherapy, radical treatment (such as radical cystectomy or radiotherapy), or palliative care. Neoadjuvant chemotherapy followed by radical cystectomy still represents the gold standard treatment for localized BC. Adjuvant chemotherapy/immunotherapy might be necessary in cases of local recurrence or metastasis [4].

Prostate cancer is the second most commonly diagnosed cancer and the sixth leading cause of cancer death among men worldwide, with an estimated 1,276,000 new cancer cases and 359,000 deaths in 2018. The global prostate cancer burden is expected to increase to nearly 2.3 million new cases and 740,000 deaths by 2040, primarily due to population growth and aging [2]. According to the latest WHO data published in 2020, prostate cancer related deaths accounted for 1.05% of total deaths in Romania [5]. The continuous progress in prostate cancer treatment options has contributed to a decrease in cancer-related deaths, even in metastatic scenarios, with a 5-year survival rate of 34% for metastatic disease [6]. PSA-based screening remains the most commonly used method, with an elevated PSA level being the most frequent indication for a prostate biopsy. There are various treatment options available for localized stages, with radiotherapy and surgery (radical prostatectomy), yielding the most effective outcomes. In metastatic cases, androgen deprivation therapy is recommended, either through surgery (bilateral orchiectomy) or medication (hormonotherapy). [7]

Tumor collision represents the encounter of two separate tumors from different topographical sites. The presence of both prostatic adenocarcinoma (PCa) and urothelial carcinoma is not uncommon in patients who undergo radical cistoprostatectomy [8]. However, cases of metastatic lymph node collision are rare, with less than 10 cases being reported in the literature. We present a case of a synchronous prostatic adenocarcinoma and MIBC with metastatic lymph node collision and a review of the currently existing literature.

## 2. Case Report

A 65-year-old patient diagnosed with pT2G3 Muscle invasive bladder cancer (MIBC) (Transurethral resection of bladder tumor performed in June 2023) was admitted to the Urology department in November 2023 for radical cystectomy. The patient followed three cycles of neoadjuvant chemotherapy with Gemcitabine 1250 mg/m^2^ on 1st and 8th day + Cisplatin 80 mg/m^2^ at 21 days (last cycle October 2023).

**Figure 1:**
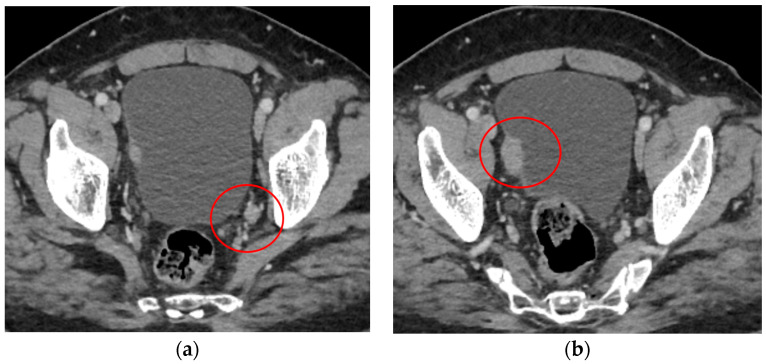
Preoperative contrast-enhanced thoraco-abdominal-pelvic CT scan. (**a**) Enlarged lymph node at the level of left obturator fossa (Red circle—enlarged lymph node). (**b**) Tumor recurrence at the level of right bladder wall (Red circle—tumor recucurrence).

The preoperative contrast-enhanced thoraco-abdominopelvic CT scan showed both kidneys of normal size with preserved renal parenchyma thickness, without hydronephrosis, with excretory function present bilaterally. The ureters were patent and not dilated. On the right side of the bladder wall, two iodophilic nodular formations of 10/9 mm and 28/16 mm were identified, with densification of the adjacent perivesical fat, suggestive of tumor recurrence. A left internal lymphadenopathy with a short axis of 10 mm was observed. (Figure 1).

The preoperative PSA value was 5.2 ng/mL. The patient presented atrial fibrillation, chronic heart failure NYHA II, diabetes mellitus, chronic kidney disease stage II KDIGO, asthma, and left hip prothesis.

**Figure 2:**
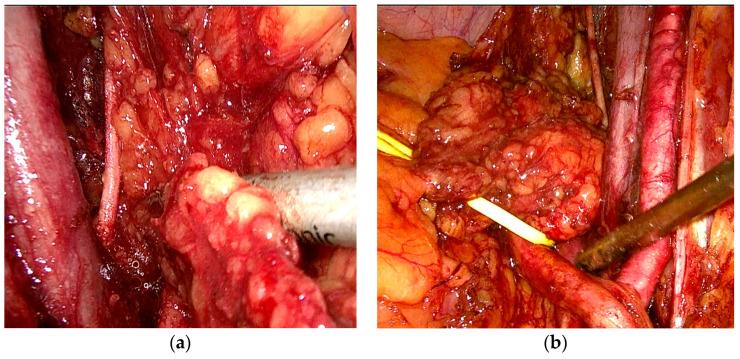
(**a**) Laparoscopic left pelvic lymphadenectomy—left obturator lymph node; (**b**) Laparoscopic right pelvic lymphadenectomy—right en bloc lymph node mass.

After discussing the case in the uro-oncological committee and presenting the therapeutic options to the patient, laparoscopic radical cistoprostatectomy with bilateral extended pelvic lymph node dissection (common iliac lymph nodes, external iliac lymph nodes, internal iliac lymph nodes, and obturator lymph nodes) and intracorporeal ileal conduit was performed. (Figure 2) The postoperative course was favorable, and the patient was discharged on the 5^th^ postoperative day. No postoperative complications were reported.

**Figure 3:**
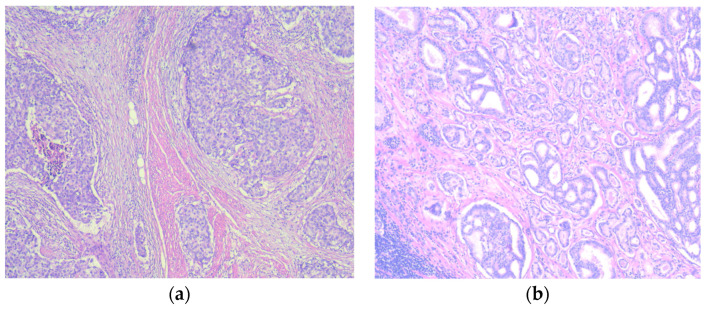
Bladder and prostate carcinomas, 100×, hematoxylin-eosin stain: (**a**) Urothelial carcinoma of the bladder demonstrating large nested architecture and infiltration of the muscularis propria; (**b**) prostatic adenocarcinoma Gleason pattern (4 + 3).

**Figure 4:**
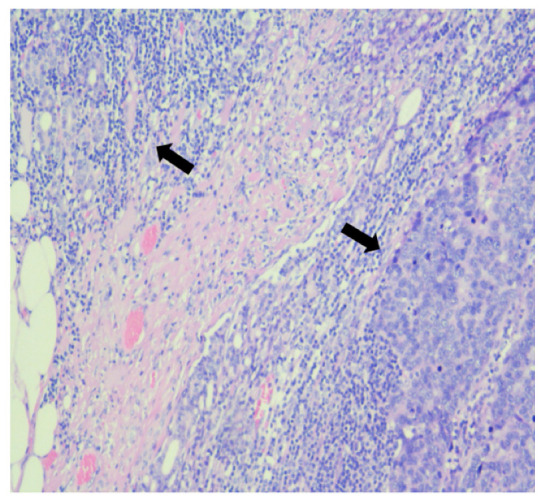
Lymph node containing two types of metastases, hematoxylin-eosin stain, 100×: prostate acinar adenocarcinoma (**top left**) and urothelial carcinoma (**right**).

The final pathology report revealed urothelial carcinoma G3 pT3bN1MxL1V1R0 as well as prostatic adenocarcinoma Gleason 7 (4 + 3) pT3bN1MxL1V0R0 that involved 24% of the gland as well as the involvement of the left seminal vesicle. Among the total of eight lymph nodes collected, one showed positivity for both urothelial carcinoma and prostate adenocarcinoma. The lymph node measured 12 mm, while the metastasis within it measured 10 mm. Immunohistochemical staining for p40, GATA3, CK19, and PSA was performed to confirm the presence of collision metastasis within the same lymph node (Figure 3 and Figure 4).

After rediscussing the case in the uro-oncological committee, the patient underwent a sequence of adjuvant radiotherapy at AL TrueBeam STx, 6x, IG-VMAT, DT-50.4Gy/28fr/42 days at the level of the urinary bladder, prostate, and adjacent pelvic lymph nodes, with a sequential boost up to DT-66Gy/37fr/55 days at the level of the bladder and prostate. Concurrently with the radiotherapy sequence, the patient underwent five cycles of chemotherapy according to the Carboplatin 2AUC protocol, under hematologic monitoring and gastric protection, with good clinical tolerance and acute toxicities (grade I digestive toxicity, grade I anemia, grade I thrombocytopenia). The postoperative PSA at one month was 1.15 ng/mL, therefore we decided to initiate hormonotherapy with leuprorelin 22.5 mg every 3 months.

**Figure 5:**
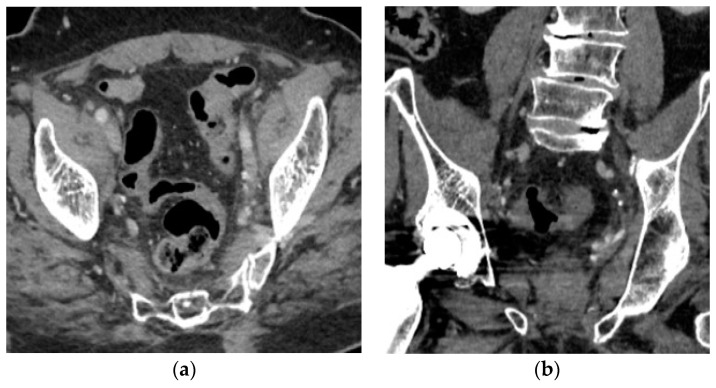
Six months follow-up contrast-enhanced CT scan: (**a**) axial view and (**b**) coronal view of the pelvis that showed no signs of recurrence or progression.

At 6 months follow-up, the thoraco-abdominopelvic CT scan showed no signs of disease recurrence or progression (Figure 5), and the PSA was undetectable.

## 3. Discussions

Prostatic adenocarcinoma finding on the final specimen after radical cistoprostatectomy is not uncommon. In a study published by Braticevici et al. [9] that evaluated the incidence of PCa finding on radical cistoprostatectomy specimens in the Romanian population, 38% of patients from 49 to 76 years presented concomitant PCa, of which 35% were clinically significant cancer (defined as Gleason Score ≥ 7 or ≥ pT2b). Saad et al. [8] revealed that out of 425 cistoprostatectomies performed in their institution, prostatic adenocarcinoma was identified in 21.2% of patients. There was no significant correlation between the level of preoperative PSA and the presence of prostatic adenocarcinoma, tumor stage, or Gleason score. A larger retrospective, multicentric study evaluated the histopathological characteristics of incidentally diagnosed PCa in cistoprostatectomy specimens. Overall, 518 (21.4%) patients presented PCa, with incidences varying significantly according to age (5.2% in those aged <50 years to 30.5% in those aged >75 years). Most of the prostate cancers were organ confined non-aggressive; however, the proportion of clinically significant PCa were significantly greater in older patients [10]. In another study realized by Abdelhady et al. [11], 20% of the patients with concomitant ADKP and MIBC presented clinically significant prostate cancer, and two patients developed local and metastatic prostate cancer recurrence.

However, collision metastasis represents the concomitant presence of two or more different tumors in the same lymph node [12]. Several physio-pathological hypotheses have been proposed to explain the phenomenon of tumor collision. It could involve the anatomical proximity of two independent primary tumors [13], an alteration of the microenvironment induced by a first tumor which favors the development of the second tumor [14], or two separate tumors influenced by a present carcinogen [15]. Moreover, this phenomenon is rare, with only few cases being reported in the literature, specifically seven collision metastases of urothelial cancer and prostatic adenocarcinoma cancer so far (Table 1).

In our case, a single lymph node was metastatic with both bladder and prostate cancer. In order to confirm the diagnosis and identify the two different metastases, immunohistochemistry with CK19, GATA3, p40, and PSA was performed. Immunohistochemistry is primarily used to differentiate prostatic adenocarcinoma from urothelial carcinoma in poorly differentiated forms. This scenario is commonly encountered in advanced prostatic tumors that infiltrate the bladder neck, as well as urothelial tumors present in the prostatic urethra. These tumors can exhibit similar morphological characteristics, especially in high-grade carcinomas, where H&E staining alone cannot identify the origin of the tumor. Furthermore, in the other cases identified, immunohistochemistry was performed in three cases using a panel that included CK7, CK20, PSA, prostatic acid phosphatase (PAP), monoclonal carcinoembryonic antigen (mCEA), and CD57. Recently, an antibody against the transcription factor GATA3 has been developed, that presents lower specificity for urothelial carcinoma but remains useful for identifying poorly differentiated tumors. Moreover, GATA3 is negative in prostate adenocarcinomas [14].

Prostate specific antigen (PSA) is the most common and relevant protein used for the management of men with suspected or diagnosed PCa. It is a serin protease produced exclusively by prostatic epithelial cells; therefore, it has a 100% specificity for prostatic tissue. However, PSA is organ specific, not cancer specific, therefore in men with suspicion of PCa it is used primarily as a screening marker. An increased serum PSA level is the most common cause for prostate cancer suspicion and subsequent prostate biopsy [21]. PSA analysis is frequently utilized in pathology as well. Given its recognized specificity to the prostate, immunohistochemical PSA analysis is regularly utilized to determine if tumors of unknown origin are linked to prostate cancer. However, in cases of poorly differentiated prostate cancers, cellular PSA expression may be significantly diminished, leading to negative PSA immunohistochemistry results and the possibility of widespread metastatic prostate cancers with very low serum PSA levels [22].

Androgen receptors (AR) such as P63 or P40 possess increased sensibility and specificity for prostatic adenocarcinoma, both low-grade and high-grade, and are less expressed in urothelial carcinomas. P63 has been primarily used to identify PCa but recent studies demonstrated similar sensibility of p40 with superior specificity [23].

Metastatic collisions within lymph nodes originating from two carcinomas from distinct topographic sites are exceptionally rare [12]. In our literature review, we identified 30 cases of metastatic lymph node collisions, including seven cases of metastatic collisions involving urothelial carcinoma and prostatic adenocarcinoma. We utilized the search term ‘collision metastasis’ combined with relevant cancer types such as ‘prostate cancer’ and ‘bladder cancer’, as well as other potential malignancies. This search was performed in the PubMed database, covering the period from January 1969 to May 2024. We included all reported cases of collision metastasis found in the literature to provide a comprehensive overview of this phenomenon (Table 2).

The prognosis of patients with collision metastases varies significantly depending on several factors, including the types of tumors involved, their respective aggressiveness, the extent of metastasis, and the overall health of the patient [19]. The heterogeneity of the tumors can complicate the diagnosis and influence the effectiveness of standard therapies, potentially leading to a poorer prognosis [12]. The nature of the primary tumors plays a crucial role. For instance, if both tumors are highly aggressive and prone to metastasize, the prognosis is generally worse. Due to the rarity of collision metastasis, comprehensive statistical data is limited. However, existing reports suggest that the prognosis can be poor, especially if the tumors are detected at an advanced stage or if there is significant metastasis beyond the initial collision site. Conversely, if the tumors are less aggressive, the patient is oligometastatic and the metastasis are associated with reduced diameter, the prognostic might be favorable.

In our case, the presence of concurrent bladder and prostate cancer may influence the choice of adjuvant therapies, such as radiotherapy, hormone therapy, or chemotherapy. Treatment decisions may need to be adjusted to address both the bladder and prostate cancers effectively while minimizing adverse effects and optimizing outcomes. Due to the small number of cases, there is no standardization of the optimal treatment approach in these cases. In a similar case, Sellman et al. [20] opted for immediate adjuvant hormonotherapy associated with chemotherapy with Gemcitabine and Cisplatin. At initial follow-up, there was no sign of progression of the disease. In another case, Junca et al. [14] opted for adjuvant therapy at the moment of disease recurrence. During the second paraclinical follow-up, a rise in PSA to 6.01 ng/mL was observed as well as multiple metastatic bone lesions on the bone scan. Additionally, a pelvic CT scan confirmed the progressive recurrence of urothelial carcinoma of the ureter. Adjuvant hormonotherapy with chemotherapy combining six cycles of Cisplatin and Gemcitabine was initiated. This treatment resulted in stabilization of the lesions at the 6-month follow-up.

We discussed our case in a multidisciplinary team, and due to our patient’s comorbidities and altered renal function, we decided to initiate adjuvant radio-chemotherapy with Carboplatin adapted to the renal function as well as hormonotherapy with leuprorelin 22.5 mg every 3 months. The follow-up consisted of monitoring the PSA level in order to detect a biochemical recurrence and thoraco-abdominopelvic CT scan every 3 months. At the 6-month follow-up, there was no imaging sign of recurrence or disease progression, and the PSA value was 0.03 ng/mL.

## 4. Limitations of the Study

The limitations of the study include the short-term follow-up period (6 months post-operatively), the lack of data regarding the treatment approach in some cases as well as the lack of detailed data regarding patient outcomes during follow-up, and the absence of information on potential complications. Additionally, the small sample size and the variability in treatment approaches make it challenging to draw definitive conclusions.

## 5. Conclusions

Collision metastasis represents a rare scenario, which might be of interest for future research. Investigating the underlying molecular mechanisms driving collision metastasis in lymph nodes could provide important insights into tumor biology and metastatic progression. The limited number of cases reported in the literature, the variability of the results and the absence of long-term follow-up make it challenging to establish a definitive prognosis for patients with collision metastasis.

## Figures and Tables

**Table 1:** Literature reports of urothelial and PCa collision metastasis occurring in the same lymph node.

	Age	Preoperative PSA Level (ng/mL)	PCa Gleason Score	Histological Type of Bladder Cancer	Antibodies Used in Immunochemistry
Ergen et al., 1995 [16]	67	10	8 (4 + 4)	High grade urothelial carcinoma	Not specified
Gohji et al., 1997 [17]	78	1.2	Not specified	Epidermoid carcinoma	PSA
Overstreet et al., 2001 [18]	67	Not specified	7 (3 + 4)	High grade urothelial carcinoma	CK20, CK7, PSA, PAP, mACE, CD57
Bhavsar et al., 2012 [19]	83	Not specified	8 (4 + 4)	High grade urothelial carcinoma	Pan-CK, CK7, CK20, PSA
Junca et al., 2015 [14]	61	10	7 (3 + 4) and ductal differentiation	High grade urothelial carcinoma	CK7, CK20, PSA, GATA3, PIN-Cocktail, RA
Sellman et al., 2018 [20]	73	26	8 (4 + 4)	High grade urothelial carcinoma	CK7, CK20, p40, PSA
Our case	64	5.2	7 (4 + 3)	High grade urothelial carcinoma	CK19, GATA3, p40, PSA

**Table 2:** Cases of collision metastasis reported in the literature.

Author, Year	Age, Sex	Primary Tumors	Nodal Site
Ergen et al., 1995 [16]	67, M	Prostatic adenocarcinoma/Urothelial bladder tumor	Pelvic
Gohji et al., 1997 [17]	78, M	Prostatic adenocarcinoma/Epidermoid bladder tumor	Pelvic
Overstreet et al., 2001 [18]	67, M	Prostatic adenocarcinoma/Urothelial bladder tumor	Pelvic
Bhavsar et al., 2012 [19]	83, M	Prostatic adenocarcinoma/Urothelial bladder tumor	Pelvic
Junca et al., 2015 [14]	61, M	Prostatic adenocarcinoma/Urothelial bladder tumor	Pelvic
Sellman et al., 2018 [20]	73, M	Prostatic adenocarcinoma/Urothelial bladder tumor	Pelvic
Our case, 2024	64, M	Prostatic adenocarcinoma/Urothelial bladder tumor	Pelvic
Sanguedolce et al., 2022 [12]	82, M	Melanoma/Urothelial bladder tumor	Pelvic
Pastolero et al., 1996 [15]	41, M	Papillary thyroid carcinoma/Medullary thyroid carcinoma	Cervical
Guelfucci et al., 2004 [24]	51, M	Papillary thyroid carcinoma/Squamous tongue carcinoma	Cervical
Elias da Cruz Perez et al., 2008 [25]	57, M	Squamous oral carcinoma/Thyroid carcinoma	Cervical
Lim et al., 2008 [26]	47, M	Papillary thyroid carcinoma/Squamous oral carcinoma	Cervical
Mattioli et al., 2009 [27]	50, F	Papillary thyroid carcinoma/Squamous cell carcinoma (unknown primary tumor)	Cervical
Zeng et al., 2012 [28]	49, F	Papillary thyroid carcinoma/Ductal breast carcinoma	Cervical
Sadat Alavi et al., 2012 [29]	32, M	Papillary thyroid carcinoma/Medullary thyroid carcinoma	Cervical
Alhanafy et al., 2016 [30]	73, F	Papillary thyroid carcinoma/Squamous thyroid carcinoma	Cervical
Xu et al., 2018 [31]	63, M	Papillary thyroid carcinoma/Squamous oral carcinoma	Cervical
Morgan et al., 1969 [32]	72, M	Prostatic adenocarcinoma/Rectal adenocarcinoma	Perirectal
Wade et al., 2004 [13]	80, M	Prostatic adenocarcinoma/ Colonic adenocarcinoma	Mesenteric
	61, M	Prostatic adenocarcinoma/ Rectal adenocarcinoma	Perirectal
Mourra et al., 2005 [33]	70, M	Prostatic adenocarcinoma/ Rectal adenocarcinoma	Perirectal
Miyauchi et al., 2013 [34]	82, M	Prostatic adenocarcinoma/ Rectal adenocarcinoma	Perirectal
Abass et al., 2015 [35]	60, M	Prostatic adenocarcinoma/ Rectal adenocarcinoma	Perirectal
El-Gendy et al., 2008 [36]	51, F	Esophageal adenocarcinoma/Ductal breast carcinoma	Thoracic
Gasparinho et al., 2011 [37]	55, F	Neuroendocrine rectal carcinoma/Ductal breast carcinoma	Thoracic
Terada et al., 1993 [38]	83, M	Prostatic adenocarcinoma/Gastric adenocarcinoma	Para-aortic
Sughayer et al., 2009 [39]	62, F	Serous papillary ovarian carcinoma/Ductal breast carcinoma	Axillary
Saco et al., 2018 [40]	71, M	Prostatic adenocarcinoma/Melanoma	Axillary
Morton et al., 2022 [41]	50, M	Prostatic adenocarcinoma/ Renal cell carcinoma	Retroperitoneum
Wu et al., 2024 [42]	79, M	Melanoma/Squamous cutaneous carcinoma	Peri-parotid
